# Bioremediation at a global scale: from the test tube to planet Earth

**DOI:** 10.1111/1751-7915.12399

**Published:** 2016-08-04

**Authors:** Víctor de Lorenzo, Philippe Marlière, Ricard Solé

**Affiliations:** ^1^ Systems Biology ProgramCentro Nacional de Biotecnología (CNB‐CSIC)Campus de CantoblancoMadrid28049Spain; ^2^ GénopoleEquipe Xénome91030Evry CedexFrance; ^3^ ICREA‐Complex Systems LaboratoryUniversitat Pompeu Fabra08003BarcelonaSpain; ^4^ Santa Fe Institute1399 Hyde Park RoadSanta FeNM87501USA

## Abstract

Planet Earth's biosphere has evolved over billions of years as a balanced bio‐geological system ultimately sustained by sunpower and the large‐scale cycling of elements largely run by the global environmental microbiome. Humans have been part of this picture for much of their existence. But the industrial revolution started in the XIX century and the subsequent advances in medicine, chemistry, agriculture and communications have impacted such balances to an unprecedented degree – and the problem has nothing but exacerbated in the last 20 years. Human overpopulation, industrial growth along with unsustainable use of natural resources have driven many sites and perhaps the planetary ecosystem as a whole, beyond recovery by spontaneous natural means, even if the immediate causes could be stopped. The most conspicuous indications of such a state of affairs include the massive change in land use, the accelerated increase in the levels of greenhouse gases, the frequent natural disasters associated to climate change and the growing non‐recyclable waste (e.g. plastics and recalcitrant chemicals) that we release to the Environment. While the whole planet is afflicted at a global scale by chemical pollution and anthropogenic emissions, the ongoing development of systems and synthetic biology, metagenomics, modern chemistry and some key concepts from ecological theory allow us to tackle this phenomenal challenge and propose large‐scale interventions aimed at reversing and even improving the situation. This involves (i) identification of key reactions or processes that need to be re‐established (or altogether created) for ecosystem reinstallation, (ii) implementation of such reactions in natural or designer hosts able to self‐replicate and deliver the corresponding activities when/where needed in a fashion guided by sound ecological modelling, (iii) dispersal of niche‐creating agents at a global scale and (iv) containment, monitoring and risk assessment of the whole process.

## Introduction

The last 20 years have witnessed an accelerated deterioration of the biological and geochemical cycles that sustain the functioning of the biosphere, a conspicuous proxy of it being the growing levels of atmospheric CO_2_ (and other greenhouse gases) and the ensuing global warming (Rogelj *et al*., 2016). There is no question that human activities, in particular the burning of fossil fuels and urban/industrial emissions have contributed decisively to this scenario, the solution (even the diagnosis) of which is by no means trivial. Several major problems have been identified, including (among others) the increasing fragility of key ecosystems experiencing both anthropogenic and climate warming‐related stresses (Barnosky *et al*., 2012). Importantly, it has been predicted that the future unfolding of these pressures will trigger sudden shifts leading to catastrophic biodiversity decays (Scheffer *et al*., 2001; Scheffer, 2009). Similarly, environmental pollution caused by man‐made (or man‐mobilized) molecules adds to this alarming stage by disturbing the ecology of a large number of ecosystems and trophic chains in an unpredictable fashion (Webster *et al*., 2014). Many recalcitrant chemicals released to the environment have become global pollutants, some of them with straight biological activities (e.g. pesticides, antibiotics, endocrine disruptors). Others crash essential natural sequences, for example, the effect of microplastics (Galloway and Lewis, 2016; see https://goo.gl/QFebmb) in feeding and reproduction of marine animals (Sussarellu *et al*., 2016; Lönnstedt and Eklöv, 2016). Paradoxically, the problems stemming from synthetic chemistry occur at a time of large lignocellulosic waste from intensive agriculture (itself fostered by man‐made fertilizers) and lack of realistic alternatives to the petroleum‐based economic growth. Overpopulation, overfishing, urban growth, expanding farming and habitat loss/fragmentation put in still more pressure on Earth's maintenance, particularly through multiple synergistic interactions among them (Newbold *et al*., 2016). The distressing part is that detailed information on each of these aspects is well known and available to the public and decision‐makers. But decisive actions to contain – let alone revert the ongoing march to environmental collapse are not at all in sight. One recurrent argument is that environmental problems do exist but addressing them in earnest would take away jobs and limit growth and prosperity of those societies and economies that are on the way to industrialization. In fact, mounting evidence indicates that inaction will be immensely much costly and damaging to our biosphere and our economy (Schneider and Mesirow, 1976). The planetary boundaries that should not be transgressed have been identified (Rockström *et al*., 2009). But dealing with the pressing agenda to maintain such safe boundaries is far from well defined (Rogelj *et al*., 2016).

## From small‐scale Bioremediation to global Terraforming

Note that these concerns, albeit at a much smaller scale, are not alien to the notions that flourished for one decade (late 1980s to late 1990s) regarding the engineering of designed microorganisms for environmental release as agents for bioremediation of chemical pollution (Daubaras and Chakrabarty, 1992; Timmis *et al*., 1994). By that time, potential targets were limited to specific sites contaminated with, for example, halogenated compounds (PCBs, dioxins, haloalkanes), nitro‐organic chemicals, heavy metals and polyaromatic hydrocarbons. The effect of anti‐flame retardants and other bioactive molecules became known later along with growing concerns for non‐biodegradable plastics. Although the field of bioremediation based on genetically designed agents raised a considerable interest for some time, the complexity of the challenge, the lack of good success stories and the widespread anti‐GMO sentiment, brought the field to a standstill (Cases and de Lorenzo, 2005). But in the meantime, the dimension of anthropogenic emissions and their consequences has revealed itself as a phenomenal planetary problem. Even the most remote and inaccessible places of Earth are contaminated by our chemical products. Under these circumstances, is it possible to envision a better, prosperous future by reconnecting us with our planet? Or are we definitely swamped into the sixth great extinction of Earth history (Ceballos *et al*., 2015) in which human actions translate into irreversible impacts that hit the delicate balances that make our planet habitable?

Along with long‐term plans grounded in a sustainable economy, new scenarios have been proposed that address the problem under an engineering perspective. This includes in particular *geo‐engineering* (Vaughan and Lenton, 2011) which relies on advanced technological solutions aiming to improve carbon sequestration, modifications of Earth's albedo or large‐scale reforestation plans. All these strategies have been shown to require staggering costs and often fail to address the real challenge in space and time. Changes involving regional or global scales require a technology that somehow scales up by itself. On the other hand, interventions had to be done in a near future to avoid sudden shifts. Fortunately, the last few years have witnessed also the onset different systemic and synthetic approaches to analyse – and whenever possible reshape complex (eco)systems (Solé, 2015; Solé *et al*., 2015). We argue not only that the global impasse caused by pollution can indeed be tackled with the tools of modern science –and not only for slowing down the ongoing trend but also for restoring lost ecosystem functionalities. And it does not imply returning to a pre‐industrial society. Quite on the contrary, this emergency offers unique opportunities to develop an entirely new knowledge‐based, sustainable economy at a global scale.

**Figure 1:**
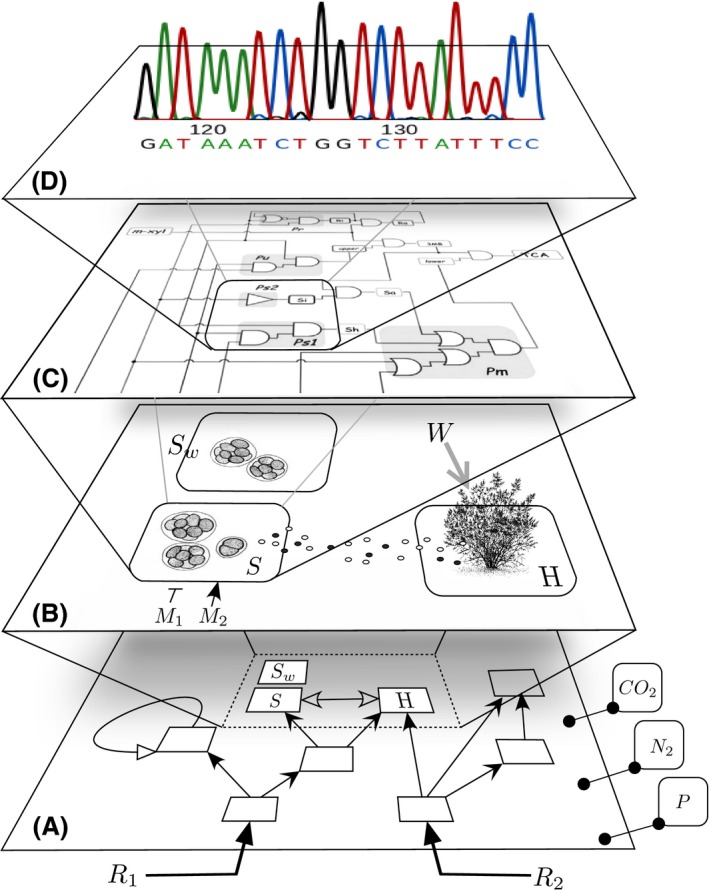
At least four scales need to be addressed for knocking‐in a new functionality within a pre‐existing ecological web: **(A)** the ecosystem level, characterized by interactions among species (S, H), external inputs/resources (R) in a biogeochemical context e.g. CO_2_, N_2_ and P availability. **(B)** the pair‐wise species interplay level, which may encompass transactions between actors of different kingdoms (microbial, plants, animals), specially mutualistic interactions, the components of which can be affected positively and negatively by external signals (M), water (W) being paramount in the whole setup. **(C)** the genetic, epigenetic and biochemical circuit level that rules the behaviour of single‐species following a logic network (the *logicome*) and **(D)** the specific DNA sequence necessary to make the biological or chemical activity of interest happen (Solé, 2015).

The key angle involves considering Earth as a feasible subject of large‐scale, self‐propagating bioremediation interventions based on the best science available. Such a view requires considering the multi scale nature of ecosystems from communities to molecular networks (Fig. 1). This endeavour echoes the planetary engineering scenarios entertained by astronomers and exobiologists under the generic designation of *Terraforming*, for example, the process of deliberately modifying the atmosphere, temperature and ecology of a planet (typically, Mars) to make it habitable (McKay *et al*., 1991; Menezes *et al*., 2015). Could not we think of developing the fundamental knowledge and the technology for *Terraforming Earth*? The theoretical possibility that engineering some key ecological interactions might do the job has been already entertained (Solé, 2015; Solé *et al*., 2015). On this background, we aim at defining a realistic baseline for a general approach to ecosystem bioengineering. This involves an unprecedented agenda that goes far beyond the classical concept of bioremediation – conceptualized as the mere removal of pollutants from given sites with biological agents. Instead, we envision a more ambitious goal that will require novel engineering perspectives and a highly interdisciplinary research effort with the global environmental microbiome at its core. In this sense, massive global metagenomic surveys (Eloe‐Fadrosh *et al*., 2016) enable the development of integrative, even trans‐kingdom metabolic models of growingly larger domains of the biosphere. This can in turn guide interventions for altering the flux of resources/elements towards a pre‐determined goal, in a fashion not very different of what metabolic engineers do today with single strains and simple bacterial consortia.

## Key reactions for restoring the balance

**Figure 2:**
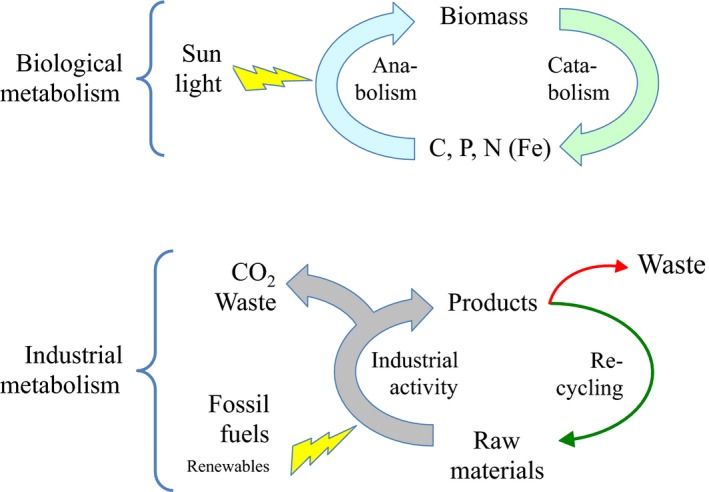
Biological metabolism versus industrial metabolism. The upper part of the figure sketches the basic metabolic cycle of the biosphere: a sustainable sequence of biochemical reactions for building (anabolism) and dismantling (catabolism) biomass and other bio‐compounds on the basis of available C, N and P species (and to a lesser extent others like Fe etc.) obtainable in the biosphere. The lower part outlines in a super‐simplified form the action of the so‐called *Industrial metabolism* (Ayres, 1994). This involves the integrated collection of human‐made physical and chemical processes that transform raw materials and (generally non‐renewable) energy into products, leaving wastes, e.g. CO_2_ and recalcitrant products and materials) along the way. The interplay between biological cycles and industrial processes at global scale was a popular topic in the late 1970s (de Rosnay, 1979). Alas, the current state of affairs makes such an industrial metabolism – and it is associated *industrial ecology* (Allenby, 2006) ultimately unsustainable. The notion of *transmetabolism* discussed in this article attempts to overcome the breach between the natural and the human‐made chemical domains by bridging the two with new reactions and rationally delivering them at a global scale by means of deeply engineered biological agents.

A large share of the problems outlined above can be traced to a number of chemical reactions involving the key elements of Life that become bottlenecks for a sustainable functioning of our natural ecosystems at both local and global scales. While defining the planetary boundaries for sustainable life, several key processes involve the existence of thresholds associated to the abundance of key molecules, including CO_2_ levels, the amount of removed atmospheric nitrogen, the quantity of phosphorous flowing into the oceans and a plethora of pollutants (Rockström *et al*., 2009). Despite the different nature of these processes, they all have in common a potentially disruptive impact that will affect global ecology in the next decades. Table 1 shows the most relevant according to the chemical species/elements involved. How can one human‐designed reaction make a difference? There are cases in our past history in which the discovery and large‐scale implementation of a new chemical transformation has been truly revolutionary, socially and economically. The so‐called Haber–Bosch reaction developed in the early XX century, through which atmospheric N_2_ is combined with H_2_ under high pressure and a catalyst, accounts for a largest share of the existing bioavailable nitrogen and it is thus responsible for modern high‐productivity agriculture (Ritter, 2008). More than one third of the World's population can be fed thanks to the Haber–Bosch process (Erisman *et al*., 2008). Other game‐changing reactions (both beneficial and detrimental) include synthetic penicillins (and other synthetic antibiotics), polythene (and other plastic polymers), contraceptive pills and liquid crystals (http://goo.gl/tUQh73). We now need many others to fulfil the remediation agenda of Table 1. The global‐scale, human‐engineered (although somewhat non‐anticipated for) *anabolism* of biological and non‐biological Earth's elements and raw materials (organic and inorganic) brought about by the chemical industry needs to be balanced by an equally man‐engineered global *catabolism* of the corresponding products (and their waste) back to become again substrates (Fig. 2). And this requires much more than petty, good‐willed recycling, but major top‐down environmental interventions.

**Table 1:** Major reactions undergone by life‐supporting chemical elements that affect the global balance of the Earth's cycles

Chem species	Status	Issues	Remediation agenda
C	C acts at the surface of the Earth in two major roles. As the atomic component with most versatile connecting capability, it enables the construction and propagation of biomatter through genetically programmed formation of C‐C, C‐O, C‐H, C‐N and C‐S bonds. As a volatile element it cycles in the atmosphere, hydrosphere and Earth crust under redox forms ranging from fully reduced (CH_4_ and coal) to fully oxidized (CO_2_), entailing major planetary consequences because of their greenhouse and ocean acidification effects	Energy vehicle Hydrocarbons Carbon fixation at all redox levels Plastics	Diversify carbon chemical reaction mechanisms by implanting in metabolism NTN^a^ catalysts of bond formation and exchange (e.g metathesis, sp2 carbon exchange, and hydroxylation, conversion of C‐H into C‐OH) so as to enable biosynthetic and bioenergetic designs not explored during evolution as well as to provide fine chemistry with chemo, regio, stereo‐specific catalysts with unprecedented scope Construct alternatives for CO_2_, CO, CH_4_ fixation so as to curb the release of greenhouse gas generation in the chemical industry and immobilize atmospheric carbon in biomaterial sinks
N	N is a universal component of biomatter (ca 15% cell dry mass) as constituent of nucleic acids and proteins. Humans require on average one mole of daily N nutritional intake. Nitrogen is used under its reduced form (ammonia) by living organisms but can be assimilated under oxidized form (nitrate, nitrite) or even N_2_ by nitrogen fixing bacteria. Vegetal growth in agriculture is mainly limited by nitrogen availability. The invention of *synthetic fertilizers*, i.e. urea produced via the reduction in N_2_ by H_2_ into NH_3_ at high pressure and temperature by the Haber–Bosch process, is estimated to have liberated human demographics and enabled about half of human population to feed. Synthetic ammonia has resulted in increasing the total terrestrial fixation of N_2_ by ca 15%, which corresponds to ~1.5% of total industrial energy consumption (as natural gas). This industrial process releases N_2_O as an end‐product of microbial fertilizer oxidation and as an atmospheric contaminant with a greenhouse effect 700‐fold higher than CO_2_	Fixation Synthetic fertilizer Energy consumption	Deploy alternative bioprocesses of nitrogen fixation from N_2_ tolerant to O_2_ in bacteria and eucaryotes (plants and fungi) through NTN biocatalysts so as to use air as feedstock Program efficient syntrophic microbe/plant associations
P	P is a universal component of biomatter (ca 1% cell dry mass) as part of nucleic acid backbone as well as animal skeleton. Humans require on average 10 mmoles of daily nutritional P intake. It exists under its oxidized form, phosphate, in the Earth crust. As such it is not volatile, does not recycle through the atmosphere and sinks in the hydrosphere to accumulate at ca 3 μM in the oceans. A phosphorus dearth is anticipated to occur in the not so distant future, once natural deposits of phosphate in Morocco, Russia and a few rare other locations will have been exhausted for fertilizing fields at the global level	Non‐renewable Extraction from seawater	Deploy alternatives to the use of phosphorus in industrial molecular products and production processes so as to spare natural resources and prevent eutrophication Deploy sustainable processes to extract P from seawater, marine sediments and wastewater using renewable energy so as to fulfil losses through seepage from continental lands
H_2_O	The elements hydrogen and oxygen mainly intervene at the surface of the Earth as water, which itself serves as reagent in a myriad of metabolic reactions for constructing biomatter. Freshwater is a rare and precious resource.	Life support Energy cycle	Exploring novel polymeric materials with highest resolution/specification of active structure and lowest cost of bioproduction, operation and environmental impact *In situ* production or water‐capture/retention activities (e.g. hygroscopic molecules and polymers) Enhanced processing with novel ion exchange resins for desalting, adsorbing and sequestering elemental pollutants ( e.g. precious elements and heavy metals)

a NTN, new to nature.

One clear avenue is the need of dematerialization (decarbonation etc.) of global energy supplies (i.e. replacement by solar, wind etc.) and the reuse of the C released to the biosphere (including non‐degradable polymers). However, the mere recycling of existing carbon excess will just slow down, but will not solve the problem of human mobilization of CO_2_. At the same time, large‐scale conversion of matter needs to remain a central operation in agriculture, nutrition, housing and transport. To this end, we need to replace bulk chemical fertilizers and mineral petroleum by biological counterparts. This is all about programming conversion of matter in a very different fashion of what has been tried thus far. For this, we have to face the inconvenient truth that stoichiometry is going to remain the ultimate criterion of industrial efficiency. Its qualitative mastery and quantitative optimization imply the systematic search forever more specific and active catalysts. Some needed reactions (e.g. accelerating improvement in atmospheric CO_2_ sequestration) could result from deliberately boosting known biological (Antonovsky *et al*., 2016; Liu *et al*., 2016) or geological (Matter *et al*., 2016) processes. But others (e.g. efficient N_2_ fixation in the presence of O_2_, water retention in arid soils, recovery of soluble phosphate from oceans, CO_2_ irreversible immobilization) may need to be altogether invented.

## Erasing the Chemistry‐Biology border

The type of global bioremediation that we envision asks also for a new type of activity holders/deliverers that merge the power of chemical catalysis with the evolutionary, self‐reproducing and spreading abilities of microorganisms. This has two aspects. One is the need to trespass the barrier between naturally‐occurring biological metabolism (Hadadi *et al*., 2016), industrial processes and environmental chemistry, so that reactions that could be developed in a purely synthetic chemistry setup could be eventually deployed by engineered (micro)biological agents. The second aspect is the development of suitable hosts that allow such reactions to be nurtured and eventually propagated globally. In other words, fuse, formalize and federate all synthetic approaches in chemistry and biology and programming *matter without barriers* between living/non‐living objects and natural/non‐natural compounds – what we have called *transmetabolism*. But how could this agenda be brought about? On the one hand, identification of new reactions, including many with non‐biological substrates, will allow us to expand beyond the natural molecular landscape and reach out novel products, properties and processes with new, Earth‐friendly functionalities. The power of synthetic biology (SynBio) will allow such reactions to be deployed by means of heavily refactored (micro)biological agents through a range of multi‐scale application scenarios. This is in fact the main advantage of Terraforming‐like approaches based on SynBio agents in respect to non‐biological geo‐engineering. In this latter case, the transformations aimed at changing the Earth's ecosystem (e.g. geological capture of CO_2_) are designed to happen intensively in a very specific location (Matter *et al*., 2016). In contrast, microbes and the activities they carry can spread very quickly and extensively through the entire microbiome of the biosphere, as the proliferation of antibiotic resistance genes has repeatedly shown.

But how may new chemical reactions be improved or altogether created? One can envision the setup of *activity farms* i.e. Laboratories/facilities where new‐to‐nature reactions will be nurtured. Such transformations of interest will be cracked by either (i) a dynamic biological setup engineered to this end, which acts as a problem‐solving device by means of a distinct selective pressure and/or (ii) the rational implantation of one or more designed steps (*metathesis*) in existing biological platforms. From the standpoint of innovation in chemistry, it is to be expected that genetic approaches (i.e. semi‐rational design and iterative improvement through selection) will enable the procedural exploration of reaction mechanisms beyond the current categories and the extension of univocal molecular constructions far beyond the current few‐kDal range. The plethora of metabolic reactions found in natural ecosystems indeed brings a full proof of the potency of the programmable search for synthetic catalysts (Hadadi *et al*., 2016). The logical next step is now to conduct the search for genetically encoded catalysts according to the customized formats to drive multi‐scale synthetic innovation. That the components of the corresponding reactions can be genetically encoded (whether in standard or alternative genetic codes) and thus amenable to directed evolution will allow users to explore a much wider landscape of chemical reactions than standard combinatorial chemistry allows thus far.

## Towards self‐replicating niche engineers

The advantage of using live organisms as the active instruments for developing such processes is that both the reactions proper and their carriers adapt to each other over time and thus optimization involving a very large number of parameters can be achieved in a relatively short time (Arnold, 2015). In other words, by building living machines capable of self‐reproduction, we could solve the problem of scaling up the engineering strategy while avoiding staggering costs. This is not only about reactions, but about chemical refactoring their live carriers in a fashion that allows the emergence of entirely new‐to‐nature biotransformations – while endowing the resulting agents with an unprecedented level of containment (Marliére, 2009). Furthermore, generation of new informational biopolymers will be interfaced with natural or artificial genetic codes and be developed for a multitude of purposes that will supersede the currently stagnant statistical structure of block‐polymers and dendrimers. From the standpoint of innovation in biology, it is to be expected that chemical availability of new molecular players (from metabolites to gene sequences to genetic polymers) will enable the emergence of biochemical alternatives in metabolic conversions and genetic coding so as to fulfil production rates and yields as well as environmental objectives of minimal carbon imprint and maximal sustainability.

Once the reactions are implemented at small scale and hosted/replicated by specialized agents, the next step is developing the technologies for converting such properties into veritable large‐scale processes that make them attractive and cost‐effective to the industrial sector. This will ask for the birth of a new branch of industrial engineering in which the whole value chain revolves around the new catalysts, which will be engineered in turn to meet operative specifications and downstream processing needs. But note that the ambition of such new wave of microbial biotechnology operations does not stop here – whatever large they may appear in comparison to previous undertakings. Contemporary SynBio allows for the first time in the Earth's history not only *invent* biological activities which have not been available before in nature, but also their deliberate spreading through much larger, even global‐scale ecosystems. To this end, we foresee the combination of the wealth of available ecological theory with CRISPR‐based *gene drive* technologies that affords exponential proliferation of specific traits through given populations (Oye *et al*., 2014). Note that the canonical gene drive strategy needs the active agent to be genetically diploid. While virtually all bacteria are haploid, given genomic portions may acquire a transient diploidy when cells get mobile genetic elements (e.g. conjugative plasmids, transposons, phages) that carry corresponding chromosomal segments. It may thus be feasible to deliberately modifying a whole bacterial population (e.g. for the sake of spreading a beneficial trait) with CRISPR/Cas9‐based tools. At the same time, it will be imperative to control the expansion of thereby reprogrammed species. But this is not a mere trial‐and‐error endeavour: it has to be sustained by solid conceptual frames, robust computing and reliable simulations –let alone a plethora of wet and *in silico* tools that need to be developed to achieve the goal. Experimental setups for engineered ecosystems (and the effect of synthetic perturbations) can find inspiration in the simple EcoSpheres that keep endlessly a closed whole of algae, bacteria and shrimps run only by sunlight (http://goo.gl/5w0r8x). Larger‐scale model habitats e.g. the so‐called ecotrons (Roy *et al*., 2016), will be extremely useful also to the same end. The ensuing information will be invaluable to set the parameters and the bottlenecks for up‐scaling such approaches.

## Outlook

In sum, we argue that the traditional views on GMO‐based bioremediation for *in situ* cleanup of chemical waste can now be empowered and expanded globally with the conceptual and material tools of systems and synthetic chemistry and biology. These will enable the technical possibility to intervene in massive environmental problems of the sort mentioned above that affect the whole planet. We are getting here in the unknown territory of global environmental intrusions, which may not be devoid of risks. But we argue that assuming a reasonable risk is preferable to the sure disastrous effect of inaction. In fact, synthetic biology has made considerable advances in recent years on how to program microbial agents to deliver their activities only when and where needed – and the certainty of containment may be much more at hand that previously thought (Schmidt and de Lorenzo, 2012, 2016). In any case, sound risk assessment must in all circumstances accompany each of the actions suggested above. In particular, we must model and inspect the responses the microbes may make to anthropogenically‐caused global changes in biological activities. One related aspect is that the technical avenues to deal with global environmental problems entertained in this article must by no means become a pretext to keep on wrecking the Earth –as there may be solutions at hand. Quite on the contrary, the ongoing environmental crisis might become an opportunity to redefine our partnership with Nature, the only alternative being migration to another planet (http://goo.gl/IpxDa0).

To a large extent, the historical unfolding of our technological impact on the biosphere has been marked by a rather unplanned use of energy‐rich molecules while delivering massive amounts of waste materials that are damaging ecosystems, polluting the air and water and limiting the spread of healthy habitats capable of providing ecosystem services. Our technological and industrial development required such energy‐rich resources, while the maintenance of our social structures is driven by this energy flow that sustains our fragile social complexity (de Rosnay, 1979). In the future, unplanned misuse of resources is likely to trigger collapse unless we start developing a new, rational relationship with a biosphere that might require an active role from our side. And only (in quantity and activity) the global environmental microbiome has the extensive catalytic power to make a difference.

## Conflict of interest

Authors declare no conflict of interest.
